# Decoding behavior from global cerebrovascular activity using neural networks

**DOI:** 10.1038/s41598-023-30661-5

**Published:** 2023-03-02

**Authors:** Béatrice Berthon, Antoine Bergel, Marta Matei, Mickaël Tanter

**Affiliations:** Physics for Medicine Institute, INSERM U1273, CNRS UMR 8063, ESPCI Paris, PSL Research University, Paris, France

**Keywords:** Imaging techniques, Neuro-vascular interactions

## Abstract

Functional Ultrasound (fUS) provides spatial and temporal frames of the vascular activity in the brain with high resolution and sensitivity in behaving animals. The large amount of resulting data is underused at present due to the lack of appropriate tools to visualize and interpret such signals. Here we show that neural networks can be trained to leverage the richness of information available in fUS datasets to reliably determine behavior, even from a single fUS 2D image after appropriate training. We illustrate the potential of this method with two examples: determining if a rat is moving or static and decoding the animal’s sleep/wake state in a neutral environment. We further demonstrate that our method can be transferred to new recordings, possibly in other animals, without additional training, thereby paving the way for real-time decoding of brain activity based on fUS data. Finally, the learned weights of the network in the latent space were analyzed to extract the relative importance of input data to classify behavior, making this a powerful tool for neuroscientific research.

## Introduction

Making sense of neural activity is one of the overarching goals of modern neuroscience. This holds true both at the fundamental level to understand how information is encoded and exchanged between brain areas but also at the application level for the development of brain-computer interfaces. Starting in the 1950’s, a body of seminal studies have deciphered the encoding of information in primary sensory cortical areas to reveal their functional organization, such as orientation maps observed in the cat visual cortex^1^, the whisker-to-barrel pathway in the somatosensory cortex^2^ or the tonotopy in the auditory cortex^3^. Later studies have shown that other brain areas display peculiar firing patterns that encode high-level—sometimes even abstract—representations of the external world, for instance place cells in the hippocampus^4^, head direction cells in the postsubiculum^5^ or ‘face cells’ in the human inferotemporal cortex^6^. This has progressively led to the idea that behavior can be decoded from neuronal activity, in particular in the dorsal hippocampus where the firing from a limited group of neurons can be used to accurately decode a rat’s location^7^. Similarly, the fact that neurons in the postsubiculum or in the antero-dorsal nucleus exhibit attractor dynamics makes decoding of head direction signal relatively simple with a limited number of units^8^.

In all of these cases however, behavior is decoded from electrophysiological signals in highly specialized brain regions, not from the brain’s global activity. This effectively limits the range of behaviors that can be decoded because it restricts the recording of neural activity to a given modality or brain region. As the amount of informative data provided by recording technologies continuously increases—high-density electrophysiology can today record from ~ 1000 recording sites from a single electrode at once^9^, calcium imaging records the activity of hundreds, even thousands, of neurons over multiple planes^10,11^ and high-resolution fMRI can acquire 3D volumes with a high temporal frame rate^12^—it is critical to address if (and how) a variety of complex behaviors can be decoded from global brain patterns. In this framework, functional ultrasound (fUS) imaging relies on continuous ultrasensitive Doppler imaging to map the cerebrovascular changes with time in the whole brain. As such, it provides rich spatial and temporal information (100 µm, 200 ms) on blood flow^13^.

Nevertheless, it is unclear whether accurate decoding can be achieved from global vascular signals that are intrinsically slower and less spatially selective than electrophysiological recordings^14^. Unlike optical imaging/fMRI setups, the portability/low constraints of fUS does not impede spontaneous behavior such as walking, running, eating, grooming and sleeping, which largely extends the diversity of possible behaviors and associated brain states observed in a single recording session. In particular, high-quality images can now be acquired continuously for several hours in freely-moving animals concurrently with electrophysiology and behavior^15–18^. This suggests that decoding techniques may perform well on fUS data, where the high temporal content of electrophysiological recordings is replaced with the high spatial diversity of fUS signals. Interestingly, fUS signals in the posterior parietal cortex of monkeys can be used to decode movement intentions during single trials in a reach task^19^. This clearly demonstrates that vascular activity in this very region conveys meaningful information so that subsequent behavior can be accurately decoded. In this paper, we asked whether global signals encompassing distant brain structures (and even vectors of spatially-averaged activity) could be used to decode behavior in a wide range of contexts.

The richness of information (hundreds of gigabytes of data per recording session) contained in fUS frames is largely underused at present: in practice, spatial information is often averaged over anatomical regions of interests (ROI) and temporal data (thus individual events) is generally averaged to derive hemodynamic response function. This is due, in particular, to the difficulty in interpreting and analyzing such large volumes of data. To address this problem, we turned to machine learning, and more specifically to Artificial Neural Networks (ANNs), a type of logical architecture which processes an input signal via a series of linear and non-linear mathematical operations, aimed at mimicking the analytical processes in the brain^20^. With their highly powerful data processing capabilities, simple ANNs can be trained in a supervised manner to extract relevant information within large and intricate datasets and perform classification tasks that would be too complex for the human eye. We therefore hypothesized that such tools, once trained appropriately could then extract enough information from a single cerebral blood volume (CBV) temporal frame to identify unambiguously the corresponding behavior.

## Results

**Figure 1:**
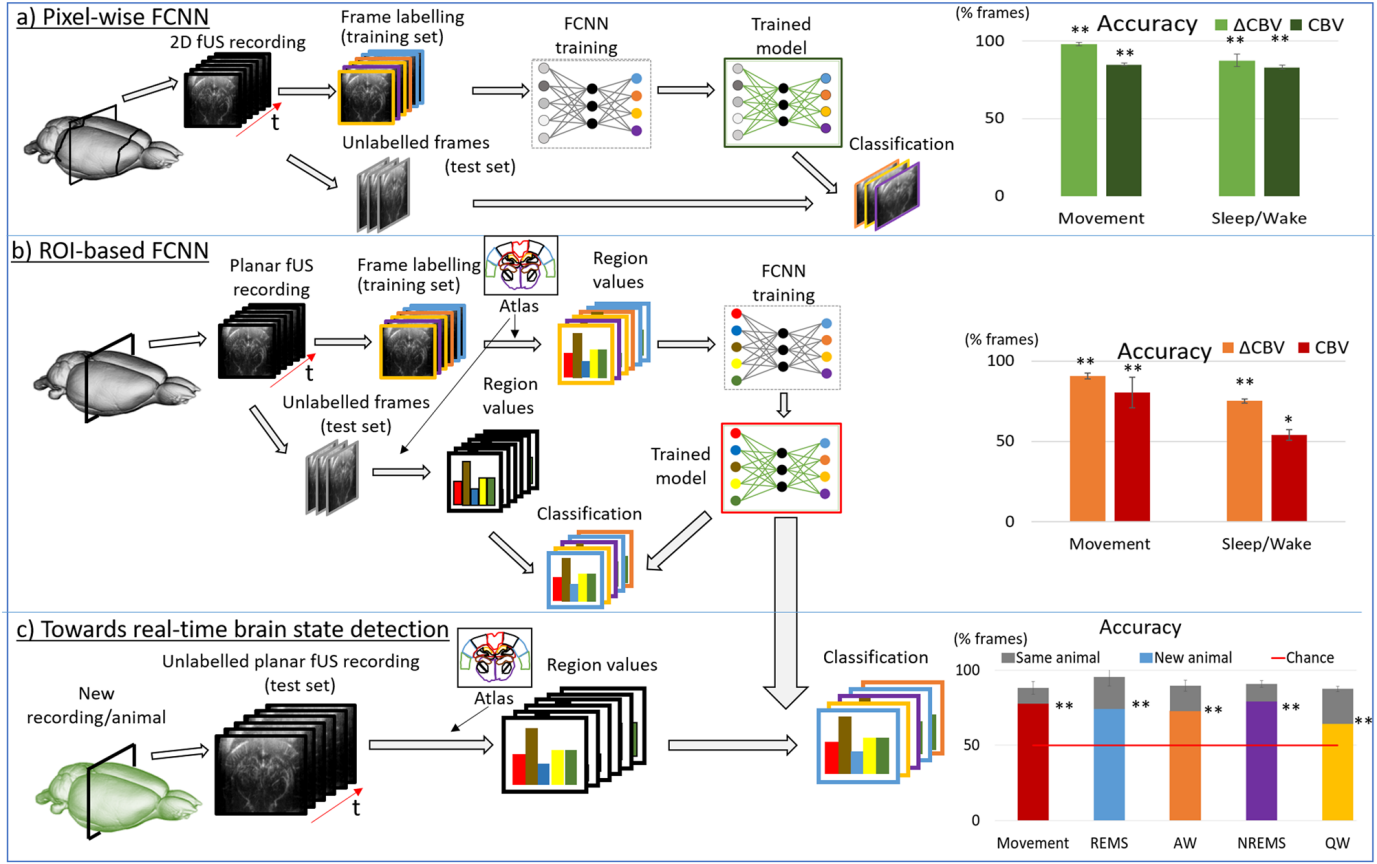
State classification pipeline scenarios. (**a**) In a first scenario, neural networks can be trained to identify from pixels values in fUS images (2500 pixels, CBV and n∆CBV) the associated behavioral states (distinction between 2 locomotion states: moving and static or 4 sleep/wake states: REM sleep (REMS), non-REM sleep (NREMS), Active Wake (AW) and Quiet Wake (QW)). The classification accuracy was above 85% for fUS images that were not normalized by a common fUS baseline (CBV) and 80% for normalized data (n∆CBV). (**b**) In a second scenario, the same decoding tasks can be performed using a much reduced information content corresponding to anatomical ROI mean values (50–80 values), obtained through expert atlas registration, with an average accuracy dropping by 13% at most for n∆CBV frames, and up to 28% for CBV frames. (**c**) In a third scenario, the second approach allowed for the classification of unseen fUS frames on any new recording (including a new animal) using the trained model providing sufficient similarity on the test and training recording sections. The corresponding bar graphs shows average accuracy values reported here as the percentage of frames adequately classified, on average across the different instances of model/animal pairs, with corresponding error bars (SD) in each case. The classification accuracy remained high even though it dropped compared to intra-animal accuracy: 78% for movement decoding and above 72% for sleep/wake state decoding (except for QW identification) as shown on the bar graph. Results of the permutation test evaluating the significance of the prediction are indicated in each case as ** (all p-values p < 0.05) or * (more than half of p-values p < 0.05).

**Figure 2:**
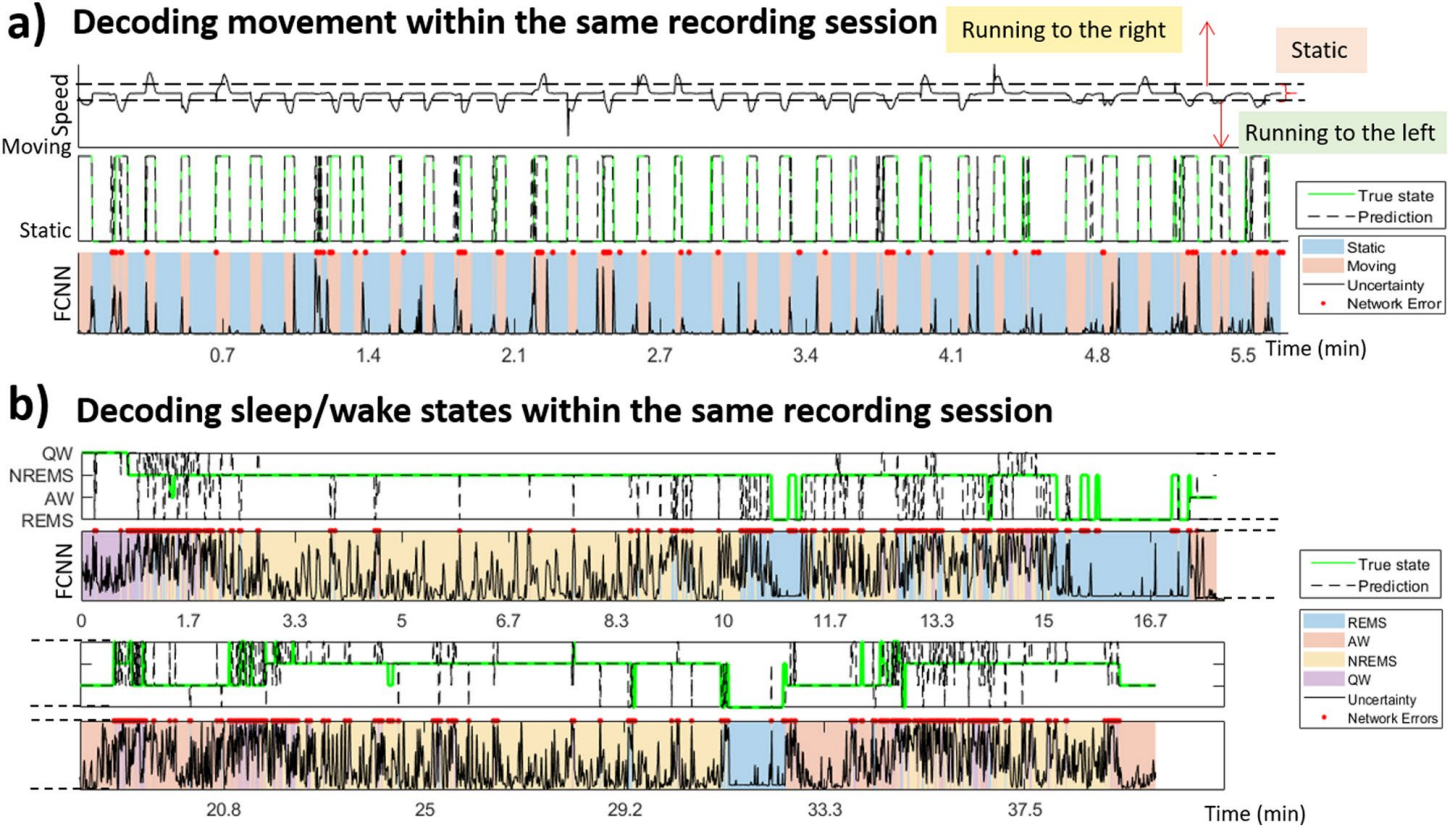
Examples of decoding within a given acquisition. ROI-based networks allowed for decoding the animal’s brain state within one recording with high performance, as shown by the temporal profiles on (**a**) (moving vs static) and (**b**) (sleep/wake states). Panel (**a**) shows the animal’s speed (top profile) and the corresponding classification labels (true state in green on middle profile). The dashed black lines on the middle profile corresponding to the networks’ prediction show excellent agreement with the true state. The bottom profile shows the networks prediction in terms of state (shaded areas are “true” states), uncertainty (black) and errors (red dots), mostly located at state transitions. Excellent agreement between the true and predicted state is also visible for the sleep/wake state decoding on panel b, except at state transitions.

In order to investigate whether ANNs could indeed decode behavior from CBV maps, we focused on one of the simplest ANN architectures: fully connected neural networks (FCNNs). In our first design, the network takes as input all the individual pixel values of fUS images acquired from a two-dimensional recording plane (cf. details in methods section, Tables 1 and 2). This results in a single network per recording with a typical input layer size of 10,000 neurons (corresponding to the number of pixels inside the 2D fUS frame, downsampled by a factor of 2. Such downsampling maintained high decoding accuracies while reducing computational load (Supplementary Fig. S1). We performed this approach on recordings from the same coronal section in 3 different rats (Antero-posterior axis: bregma = − 4.0 mm) during a simple locomotion task where animals were running back and forth for water reward on a 2.2-m long linear track (cf. “Methods”). Such a section was chosen because it allowed the simultaneous monitoring of the retrosplenial cortex, dorsal hippocampus, dorsal and ventral thalamus and hypothalamus, structures that are involved in different aspects of spatial learning. The actual position and speed of the animal on the track were used to classify frames into two categories corresponding to a moving (running) or a static state. We found that on a given acquisition, the network was able to classify on average 98% (± 1%) of the test frames into the right category (cf. Figs. 1a, 2a and Supplementary Table S1), indicating that the pixel amplitude information in a single fUS image is sufficient to determine the underlying locomotion state. We trained the FCNN using both raw ∆CBV and relative (n∆CBV) CBV profiles –obtained after pixel-wise normalization—and assessed performance the two cases. Relative CBV profiles are obtained by acquiring the first 3 min of quiet wake or any stable condition (before or during the recording) and subtracting and dividing the mean of this distribution for each pixel, to express it as a ∆F/F variable (commonly used in imaging studies where absolute values of the signal strongly differ across pixels, cf. methods section). Performance was best when using the relative CBV profile (n∆CBV) than with the raw fUS images (∆CBV), because this image normalization process facilitates the separability of the dataset. K-fold cross-validation (k = 5) showed that these results were largely robust across training instances within 2% of the accuracy value (cf. Supplementary Table S1). An example of this decoding is shown on Fig. 2b).

**Table 1:** FCNN architecture parameters, used both for sleep/wake and locomotion problems.

	Pixel-based	ROI-based
Downsampling rate	2	N/A
Masking	Yes	N/A
Input feature nb	Nb pixels/4*	Nb regions
Feature normalization	In range [− 1, 1]	
Balanced dataset	Yes	

_*__The number of input pixels is divided by 4 because of the downsampling._

**Table 2:** FCNN training parameters used for each classification problem.

	Sleep/wake		Movement
Hidden layers	1		
Hidden layer neurons	3		
Output neurons	4	2	
Output activation	Softmax		
Max epochs	10,000 (+ early stopping, cf. text)		
Optimization	Minibatch stochastic gradient descent		
Minibatch size	8		6
Initial learning rate	0.05		5
Learning rate decay	$\alpha =\frac{\alpha }{1+ep}$		
Regularization	None

We further increased the level of complexity of the classification and evaluated our approach in a more challenging context, aiming to distinguish between 4 different sleep/wake states, namely quiet wake (QW), active wake (AW), non-REM sleep (NREMS) and REM sleep (REMS) (cf. Fig. 2c). Previous work have shown that vascular activity during REMS strongly differs from other states, but NREMS and QW show very similar profiles^17^. fUS images were obtained for 6 animals over the same coronal section (AP axis bregma = − 4.0 mm) during long recordings where animals spontaneously alternate between periods of sleep and wake. The data were labelled via traditional sleep scoring derived from accelerometer/Local Field Potential (LFP)/Electromyographic (EMG) recordings (cf. methods section). We found that on a given acquisition, the same network architecture was able to classify more than 87 ± 4% of fUS frames into the right brain state, when using the whole fUS image (pixel-based approach). Precision (proportion of labels detected by the network which are correct) and Recall (proportion of true labels detected by the network) measurements for each state showed that REMS was the easiest state to distinguish, followed by NREMS and AW, while lower Recall and Precision values (although still above 80%) were obtained for QW, which was expected (cf. Supplementary Table S2).

To extend the capabilities of our methodology, we registered the Paxinos atlas^21^ onto fUS images to locate anatomical regions of interests (ROIs) and extract mean values of the CBV for each temporal frame within these anatomical ROIs. This results in generating an input vector for each frame which size is the number of ROIs within the corresponding recording plane and we trained a second network architecture using these vectors as inputs (Fig. 1b). With such architecture (cf. details in methods section, Tables 1 and 2), decoding of the locomotion state on 2D fUS images and atlas registration was achieved with an average accuracy of 93 ± 1% (Fig. 1c) for n∆CBV inputs. For the identification of sleep/wake states, this ROI-based approach was able to accurately classify more than 75 ± 1% of fUS frames into one of the 4 states for n∆CBV data.

The ROI-based approach described above allows for the application of a previously trained network to “novel” recordings, in particular in a different animal, and therefore makes real-time decoding of behavior possible, since all preprocessing steps as well as the network classification time are almost instantaneous (1 ms), though we do not demonstrate it here. Using this approach, we evaluated if information learnt by the network on one animal could be applied blindly to n∆CBV patterns acquired on a previously unseen animal (cf. Fig. 1c). We found that our decoding method was transferable with little loss of performance to a different animal as it was capable of accurately decoding the locomotion state of a previously unseen animal with an accuracy of 78 ± 8%, losing only 14% accuracy to the transfer to a new recording. For the identification of sleep/wake states, REMS, NREMS, AW and QW were treated independently, as we evaluated the ability to identify each state among other sleep/wake states. We found that our method could identify REMS, AW and NREMS on a previously unseen animal with 74 ± 4%, 72 ± 2% and 79 ± 2% accuracy respectively. Lower accuracy was reached for QW. This was obtained for 5 recordings for which anatomical coordinates of the reference and unseen recording could vary by as much as 0.7 mm, but contained the same anatomical structures, which likely explains the drop in accuracy observed.

**Figure 3:**
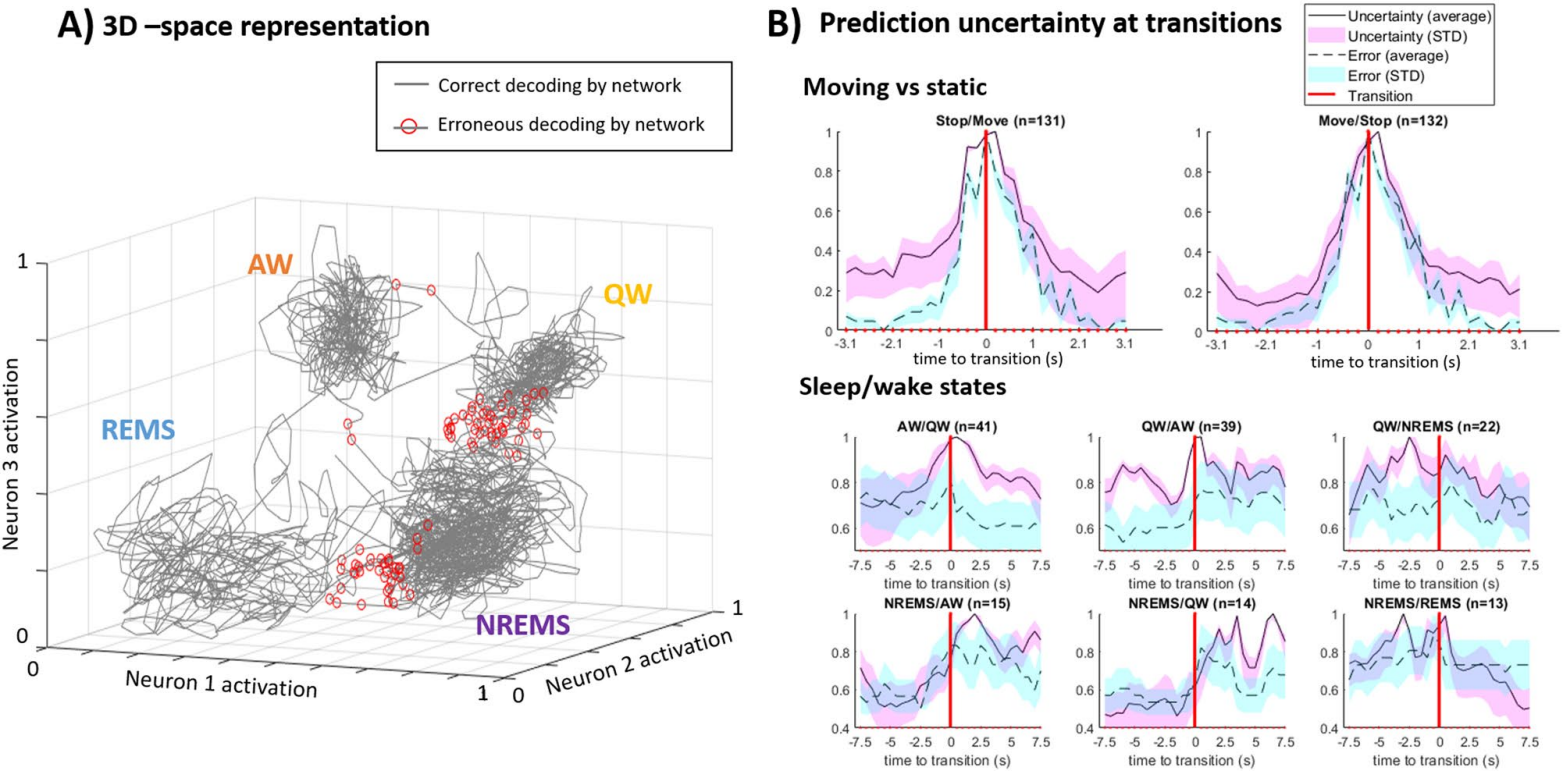
Visualization of the decoding error and uncertainty in time and space. (**A**) The architecture of our networks with a hidden-layer of dimension 3 allows for a visualization of the network’s activation in the latent space in 3D, here in the case of sleep/wake state identification based on pixel values. fUS frames labelled with the same sleep/wake state are grouped together in this state, and errors (red circles) are located at boundaries between the 4 different clusters, suggesting that they occur mainly at state transitions. This is confirmed on (**B**), showing consistently higher prediction uncertainty values and error rates near state transitions for the binary locomotion and for most of the sleep/wake transitions in the state decoding tasks (n represents the number of such transitions available in the data). Uncertainty of the network rises from 1 to 2 s to a state transition, which is consistent with the delays of neurovascular coupling. For NREMS/AW and NREMS/QW the uncertainty and error rate peak after the actual transition and remain high several seconds afterwards, which may be attributed to the “progressive” nature of these transitions.

To understand how the neural networks could perform so well, we looked into the weights learnt to separate the different states. The fact that the hidden layer comprised just three neurons allowed for a visualization of the input data in the latent space in 3D and asses how the neural network algorithm is able to provide maximal separation between states. Figure 3A shows that by plotting each classified fUS frame in this hidden-layer (latent) space (using hidden neurons’ activation as x, y and z coordinates), the data appears in the form of clusters of various shapes corresponding to the different sleep/wake states. Some clusters, like the one corresponding to AW, appear more compact, which indicates a high similarity of fUS frames in this state. Conversely, this 3D topology could highlight the potential presence of sub-states within one given cluster. It can also be noted that some clusters, like AW and REMS, are more distant than others in this space, meaning a lesser degree of similarity and a higher separability of the associated cerebrovascular activity patterns. This representation can be applied to a sequential visualization of the animal’s natural consecutive sleep/wake states in space (cf. Supplementary video S1).

Interestingly, classification errors (indicated as red dots on Fig. 3A) occur specifically at cluster boundaries nearest other clusters. More specific analysis found that in the case of sleep/wake states identification, 65% of the errors occur within 47 s of a transition between states. (Supplementary Fig. S3b). This confirms that the network makes most of its errors at transitions between states, which in the case of sleep are not sharp, and thus intrinsically difficult to define^22^. The classification errors observed could arise from the similarity between the spatial patterns exhibited in different states, making it difficult for the network to classify, but could also be due to incorrect labelling which is more prone to occur at transitions. This held true for locomotion states (moving vs static) where 76% of errors were located within 1.5 s of a transition (Supplementary Fig. S3a). The larger temporal span of classification errors obtained in the sleep/wake task (see above) compared to this task can be explained by numerous factors including more blurred transitions as opposed to sharp transition for the locomotion states, increased errors due to multiple states, incorrect sleep scoring on microstates like micro-arousals or micro-REMS episodes. We then investigated if the network’s errors could inform us on a given state change. In our architecture, the network’s last layer provides probability values for the different possible output categories, before choosing the one with the highest probability as a final output. We defined the uncertainty of the network, as the difference in network output value between the predicted class (highest probability) and the second most probable class and found that the uncertainty was particularly high at transitions. Panel (b) of Fig. 3 shows that the prediction uncertainty increases by about 60% within 1–2 s of a transition between movement and static states. In addition, we found that 80% of the cases for which the network uncertainty rose by 60% or more happened within 2 s of a state change. Thus, despite the late response of fUS signals due to the delayed hemodynamic response function, a 60% rise in uncertainty could be used to anticipate changes between motion and immobility with an accuracy of 80%. For sleep/wake state identification, a 60% rise in uncertainty values happened within 41 s of a state change for 76% of state changes (cf. Supplementary Fig. S3b). A 60% rise in uncertainty of the network’s prediction could therefore be used as a metric to predict changes between sleep/wake states in real-time with a 76% accuracy in this case. Uncertainty profiles for transitions towards a wake state, and for the transition from AW to NREMS show a clear uncertainty peak after the transition as determined by traditional sleep scoring, which indicates transitional periods during which cerebrovascular patterns are rearranging. Interestingly these time delays (namely 1.5–2 s) correspond to the ones of neurovascular coupling traditionally observed with fUS, confirming that sharp transitions between well-defined electrophysiological profiles (like from NREMS to REMS) are mirrored in cerebrovascular patterns with a time constant compatible with physiological processes.

**Figure 4:**
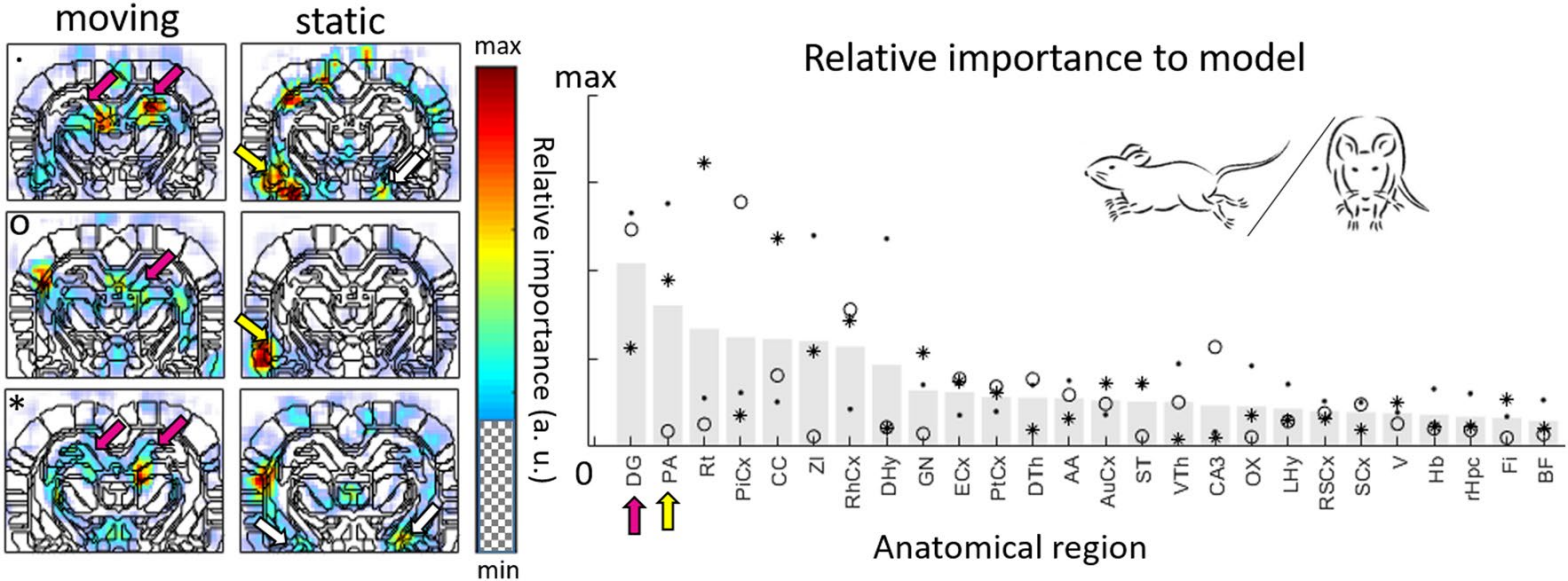
Importance to the classification of different anatomical ROIs for the detection of the locomotion state (animal static or moving). The network’s learnt weights allowed for the visualization of spatial maps of relative region importance to the classification. Values provided on the graphs, corresponding to the relative importance of ROIs in ROI-based classification, as provided by the Holdback Input Randomization method, are averaged across the three animals with data points shown in different black markers for each animal. The 2D-maps displaying the local relative importance at bregma = − 4.0 mm show, on all of the three recordings considered (three different animals), high importance of the dentate gyrus (DG) for identifying the moving state, and a high importance of the central region of the ventral thalamic nucleus region and posterior amygdala (PA) region for the static state, confirming the ROI-based analysis.

We finally investigated whether the weights learnt by the network could inform on which pixels or anatomical regions were more important than others to classify states. This was done using the Holdback Input Randomization method^23^, which consists in evaluating the drop in accuracy when removing a given input (in our example, a region or pixel value) from the input to the network. This can then be displayed using an appropriate anatomical atlas as a spatial map specific to any of the network’s output node, corresponding to a given behavior (‘moving’ for instance). In the case of ROI-based networks, this technique provided a ranking of the different input ROIS with respect to their importance to the classification task, whereas 2D-importance maps were provided in the case of pixel-wise networks. Figure 4 shows the results obtained for classification between static and moving states, where the ROI found most important to the classification was the Dentate Gyrus in the dorsal hippocampus, which is critical for the processing of spatial information^24^. Regions present independently on both hemispheres were grouped for the ROI-based analysis only, but a symmetry is nevertheless visible on the corresponding maps obtained from the pixel-wise analysis.

**Figure 5:**
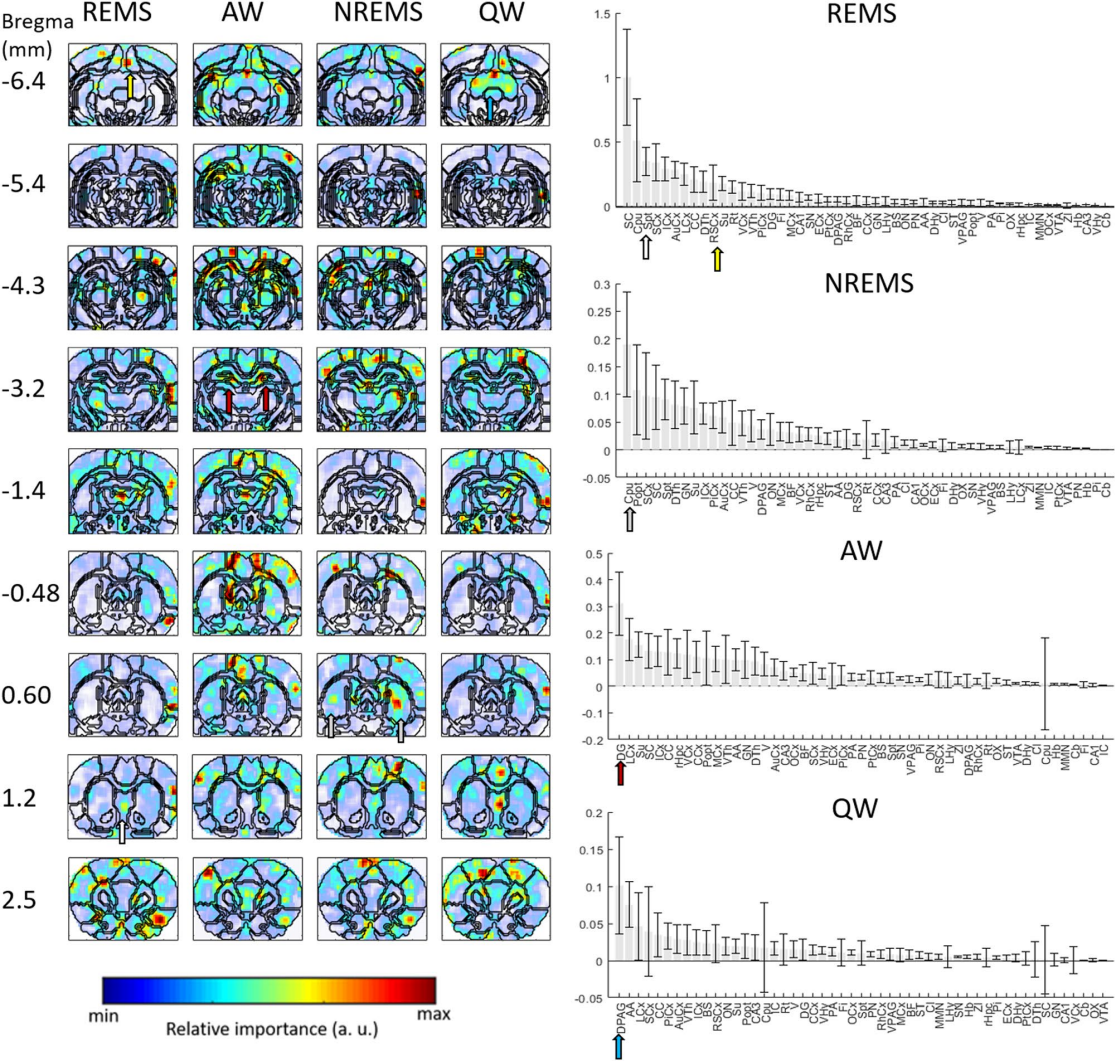
Importance to the classification of different anatomical ROIs for sleep/wake states across the brain. Neural networks were trained pixel-wise and ROI-wise on 9 different coronal planes spanning the brain between bregma -6.5 mm and bregma 2.5 mm. Relative importance of anatomical regions calculated across those 9 planes are presented here for each sleep/wake state. Corresponding pixel-wise importance maps confirm the ROI-wise findings, and bring additional local information. For example, the contribution for REMS of the azygos anterior cerebral artery (azac), which does not correspond to a single ROI in our method is only visible on the pixel-wise maps. In the case of AW, the dentate gyrus (DG) and whole Hippocampus (whole Hpc) to a lesser extent are clearly visible on the pixel-wise map. For NREMS, the caudate putamen (CPu), which stands out as the most important ROI is highlighted on the pixel-wise map, in particular its central area. The map for QW shows highly heterogeneous importance within regions of the Periaqueductal gray (PAG), somatosensory cortex (SCx) and superior colliculus (SC). This is in line with the largely different importance calculated for the ROIs of the PAG (dorsal and ventral) and of the Colliculus. The region of the piriform cortex also appears highlighted on the pixel-wise map.

For sleep/wake state identification, the same approach and corresponding spatial maps were obtained for 9 coronal planes throughout the brain (Fig. 5 and Supplementary Fig. S2). The corresponding pixel-based and ROI-based networks reached training accuracies of 96% and 77% respectively on average across all coronal planes. Individual analysis highlighted a number of ROIs most important for the identification of each sleep/wake state. For REMS, these were the superior colliculus, which has been shown to play a role in wakefulness induction and is known to be involved in the visual processing of dream content^26^, the striatum and medial septum. These regions, as well as the retrosplenial cortex and the azygos anterior cerebral artery (azac), were also clearly highlighted for REMS on pixel-based maps, which are strongly activated during REMS^27^. For AW, regions clearly highlighted by both ROI-based and pixel-wise analysis were the Dentate Gyrus, which was found to be heavily involved in voluntary running^28^, and to a lesser extent the Limbic Cortex, Subiculum and residual Hippocampus and the Visual and Motor Cortex. The striatum was most evident for NREMS, and although differences were smaller, the dorsal PAG, superior colliculus and piriform cortex were also highlighted for QW. Interestingly, a ROI found to be relatively important to the classification can appear highly heterogeneous on the pixel-wise importance maps, potentially indicating a heterogeneous activity within that region, but also highlighting the complementarity of the two approaches (cf. Fig. 5).

## Discussion

A simple fully connected neural network architecture has shown that CBV patterns measured with fUS images contain enough information, despite the smaller temporal resolution of vascular signals and temporal delays due to neurovascular coupling, to perform high performance decoding of brain activity for a range of behavioral tasks, even on unseen animals. In this work, care was taken to minimize potential information leakage bias due to the slow decorrelation of the fUS signals (mutual information between temporally adjacent frames is not zero, which might positively bias the evaluation of our networks accuracy). The accuracies reported for classification within acquisitions were therefore evaluated only for test frames which were not directly adjacent to the training frames. Quantifying information leakage would yield complex analysis, but the decorrelation times of fUS signals have been estimated indirectly to correspond to about 1 s as a rough average^17,18^, which corresponds to a 2–3 frames distance in the sleep/wake case and 4–5 frames in the case of locomotion decoding. In the case of locomotion, removing these frames from the analysis would have resulted in too few frames for an informative accuracy measure (cf. Supplementary Fig. S5). However, for the sleep/wake case, supplementary analysis shows that the global performance does not decrease by more than 10% when removing more adjacent frames, except in the specific case of Sleep/Wake identification on region-based data without normalization (cf. Supplementary Fig. S4), which highlights again the importance of the normalization step. These drops in accuracy remain much below the variability of the accuracy across acquisitions (small effect size), and do not increase when choosing for testing only frames even further away temporally from the training set. Besides, the high performance obtained when decoding a previously unseen animal confirms that the fUS data content is specific and rich enough to identify a single behavioral state, even when considering only mean activations within ROIs. This methodology can be applied in theory to fUS data recorded for any setup, for the identification of any task-specific brain state as long as an accurate prior data labelling (and atlas registration in the ROI-based approach) is possible, although the network architecture and implementation details may change. In this study, results were limited to a small number of 3 to 6 animals and 9 imaging planes, and it remains to be seen how this methodology scales up to larger datasets and applies to different areas and planar views of the brain. It can already be noted that high accuracy was obtained for 9 different coronal planes in the case of sleep/wake state identification. Given the results achieved so far, we hypothesize here that a large set of recordings spanning the entire brain could allow for a readily-trained set of networks able to decode in real-time the sleep/wake or locomotion state for any given coronal fUS image (since network prediction and preprocessing steps are quasi-instantaneous). This ability of the network to classify naïvely on unseen recordings is extremely powerful and can be applied in a wide range of contexts such as brain-computer interfaces and manipulation of brain activity based on fUS signals. This has a range of potential applications, from real-time classification of the animal’s sleep/wake state during an experiment, to a posteriori decoding of the animal’s activity. At present, precise real-time decoding requires the acquisition of a baseline fUS recording so as to normalize the subsequent CBV frames accordingly to obtain n∆CBV frames. This can be done in 3 min at the beginning of any recording during a stable condition. A shorter period such as 1 min could also be used as long as mean and standard-deviation can be accurately evaluated from the sampling period. However, the accuracy for region-based training on CBV values remains much above 25% for sleep/wake states (which would indicate performance at chance level), indicating that the network can already learn to separate the different states without normalizing to a baseline. The sole use of CBV data may therefore be possible in the future to directly perform accurate real-time sleep scoring.

Careful registration to an anatomical atlas is crucial for reliable data labelling when decoding across animals. This constitutes the main limitation of our methodology and is likely to have limited the results achieved so far. As an example, decoding from one animal to the other based on all pixel values is only possible if both acquisitions are perfectly registered, and if inter-individual variability is small, leading to pixels at the same location in the images representing the same spatial location within the brain. Because our current methodology could not ascertain a registration accuracy within 1–2 pixels, we chose to focus on region-based analysis instead, which does not require the same level of precision. Yet, ever increasing registration computing capabilities^29^ and better performing and fully automatized registration algorithms^30^ will hopefully make this atlas registration readily usable, automatic and increasingly accurate. In general terms, the networks presented here were chosen for their simplicity, so as to avoid any overfitting to the data, but also an easy access to the network’s learnt information. The classification performance can be optimized in a number of ways, including larger datasets with more accurate data labelling (especially for sleep scoring) and registration, as well as higher level optimizing of the network hyperparameters.

The imaging of freely moving rodents, as performed here, permits many applications studying specific routine tasks including (but not limited to) locomotion, and the associated n∆CBV change patterns. By analogy with place cells or head-direction cells, the ability of n∆CBV frames to reliably assess motion as demonstrated here, questions the potential existence of vascular place patterns associated to locomotion, and possibly also to spatial position. Our approach may prove extremely useful in exploring the existence of such maps.

Finally, the study of the network’s uncertainty, or the projection of a given frame in the latent space of the network may provide a metric for the prediction of state transitions, and possibly new criteria for the definition of such transitions. This could be particularly adapted to applications such as sleep/wake state identification or decision-based behavior, where neurological transition between states are known not be instantaneous (and are not always sharply defined like in “intermediate” sleep) and can often precede actual motor action by a significant delay^31^. We reported results based on the assumption that the data labels represent the “true” state of the animal. However, it may be that the CBV patterns are capable of informing more precise or more accurate data labelling, and that cases identified as classification errors actually represent labelling errors or additional sub-states undetected by traditional sleep scoring for example. This is reinforced by the fact that 96% of classification errors for sleep/wake states were located in groups of 3 or more consecutive errors, as visible on Fig. 2A). Yet, the network had no information about the temporal sequence of those frames, but chose to group them based on their spatial CBV pattern.

In addition to the temporal information reconstructed by the networks, the importance of the different anatomical regions for each classification task provides information on the cerebral CBV and n∆CBV patterns specific to a given behavior. In the case of a binary classification (static/moving task decision), we cannot unequivocally associate a region to a brain state. Our method simply tells us what pixels/regions are of interest to the network to perform well. However this is not the case in non-binary classifications. Although such maps do not directly represent the relative activity of the different regions during a task, they provide large-scale vascular ‘importance maps’ associated with a specific behavior, something which is hard to achieve with actual recording techniques. In this study, our main findings were in line with previously published material. Thorough validation will be required to compare patterns produced by our method to actual physiological measurements of brain activity. However, our method can readily highlight a large number of new regions of interest for subsequent investigation using electrophysiological recordings and/or optogenetic manipulation. The possibility of acquiring fUS data on freely moving animals will allow for extensive evaluation of this potential in the future. Besides, our machine learning-based approach is not limited to studies on rodents and can be applied to the imaging of any species imaged with fUS, including humans.

## Methods

### Functional ultrasound data

#### Animal model

All methods are in compliance with the European Communities Council Directive of 2010. All experiments received ethical approval by the French government and ethical committee for the Paris Centre and South region. All methods in this study are reported according to ARRIVE guidelines.

This analysis was based on fUS recordings obtained in different studies^15,17,18^, for which 12 adult Sprague Dawley rats aged 10–12 weeks underwent surgical craniotomy under anesthesia induced with 2% isoflurane and maintained by ketamine/xylazine (80/10 mg/kg), to expose the brain from bregma + 6.0 to bregma − 8.0 mm, with a maximal width of 14 mm. There was no control group nor blinding, randomization or exclusion in this case as it was designed as a behavioral study. Specifically designed electrodes made of bundles of insulated tungsten wires were implanted stereotaxically into the brain tissue and lowered in the dorsal hippocampus at stereotaxic coordinates AP =  − 4.0 mm, ML =  ± 2.5 mm and DV =  − 1.5 mm to − 4.5 mm relative to the Bregma. These were used to record Local Field Potentials (LFP) via a 32-channel amplifier with high input impedance and a gain of 1000, DC-cut at 1 Hz, and digitized at 20 kHz (Blackrock microsystems). In this study, the outcome measures were the imaging and electrophysiology measurements. No euthanasia was used in this study.

#### fUS acquisition

Ultrasound images of the brain vasculature were obtained for all animals via ultrafast Power Doppler based on plane wave transmissions. A 15-MHz probe designed for animal studies was fixed to the animal’s skull and driven by a fully programmable GPU-based ultrafast ultrasound scanner (Inserm Accelerator in Technological Research for Biomedical Ultrasound, Paris France). Images were acquired continuously at a pulse repetition frequency of 500 Hz, with a final sampling rate of 1–2.5 Hz depending on the recording. A total of 8 plane waves were acquired for angles with the probe equally spaced between − 7° and 7°, and coherently compounded^32^ to form high quality images, at a frame rate of 500 Hz. More information about the animal surgery, LFP recordings and fUS acquisitions is available in^17^. A 3D-volume was also obtained by acquiring Doppler images for 9 coronal planes across a rat brain from bregma − 7.0 mm to bregma 3.0 mm.

#### Experiment design

For sleep scoring experiments, the animal was placed awake in a rectangular or round box and was imaged freely moving around during a period of time comprising sleeping and awake states at a sampling frequency of 1 Hz. For running experiments the animal was positioned onto a 0.2 m by 2.35 m corridor 40–60 min and was imaged during free movement along this corridor, during a period comprising running and static periods at a sampling frequency of 2.5 Hz.

#### Image analysis

To discriminate blood signals from tissue clutter, the ultrafast compound Doppler frame stack was filtered via Singular Value Decomposition^33^, removing the N = 60 first components. The Cerebral Blood Volume (CBV) frames obtained were further normalized by a baseline image corresponding to the average of the first 3 min of the acquisition, leading to n∆CBV frames. For each pixel, the mean value of the baseline distribution is subtracted and divided to obtain a ∆F/F (activity expressed as a percent of change relative to the baseline). ROIs were extracted from the Paxinos Atlas^21^ and carefully overlayed onto the fUS images using salient anatomical and vascular structures (cortex edges, sine veins, Willis polygon when visible). This led to 71 and 82 ROIs used for locomotion and sleep/wake state decoding respectively (cf. Fig. 1).

In order to apply a model trained on one acquisition to a different acquisition, only ROIs present on all acquisitions were kept within the locomotion and the sleep/wake datasets. ROIs smaller than 20 pixels on the original image were included in the closest anatomical ROI in terms of location and function. This led to 26 and 22 ROIs for the decoding of movement and sleep/wake state respectively.

For the identification of the sleep/wake state on several coronal planes of the same rat, the regions were grouped into 53 symmetric anatomical regions to keep a coherence between the planes. These regions as well as the corresponding acronyms and Paxinos regions included are given in Fig. S4 of the supplementary materials.

### Neural networks

#### Data labelling

For movement decoding, the position of the animal along the corridor was obtained via histogram thresholding of video frames acquired during the experiment and synchronized with the fUS frames. Only one coordinate was used as the range of positions along the axis perpendicular to the corridor was very small compared to the corridor length. For movement decoding, the animal’s speed along the corridor was estimated as the difference in position between two consecutive frames. In the training process, the frames for which the speed was smaller than 0.1 m/s were then labelled as static, the frames for which the speed was larger than 0.1 m/s were labelled as moving. Other frames were discarded for the training to keep both classes distinct, leading to 1% of frames rejected from the analysis on average across acquisitions.

For sleep/wake state decoding, each fUS frame was associated with a single categorical label corresponding to one of 4 different states. The sleep scoring procedure was based on traditional methods^34^ using neck electromyogram, animal movement and LFP to discriminate between active wake (AW), quiet wake (QW), NREM sleep (NREMS) and REM sleep (REMS). Sleep and wake were discriminated by applying a threshold on EMG activity. A three-dimensional (3D) miniature accelerometer placed on the head of the animal was used to discriminate between quiet wake (QW) and active wake (AW). Quiet wake was detected when the EMG was high and the animal stood still with its head close to the ground. Active wake was detected accelerometer activity exceeded a threshold, i.e. when the animal was either moving, walking, running or standing and whisking in the air. When the EMG dropped below a threshold (variable across recordings, set during offline processing) for more than 10 s, we labeled the sub-sequent period sleep. NREMS sleep is characterized by a large amplitude of irregular activity (white noise distribution between 1 and 50 Hz on the time–frequency spectrogram, high ripple activity, and low theta/delta ratio), whereas REMS is characterized by increased theta/delta ratio, minimal EMG, and decreased ripple power. A last check was the brief awakening following REMS episodes.

#### FCNN architecture

Three-layer fully connected neural networks written in custom Matlab code were used throughout this study. They comprised a variable number of input neurons (depending on scenarios described below), 3 neurons in the hidden layer and a number of output neurons corresponding to the number of categories to classify the data into. This number of hidden layer neurons was chosen as the best compromise between reduced layer complexity/absence of overfitting and accuracy, in addition to providing 3D space representation capabilities. All hidden layer neurons were used with ReLu activation and softmax activation was used in the output layer.

Two approaches were used for each classification experiment.

- Pixel-based approach: the network takes as an input all the pixels in the grayscale image. In this case, the fUS images were downsampled by a factor of 2 using a max-pooling algorithm with kernel size 2 × 2 and a stride of 2 in both vertical and horizontal directions, so as to reduce the dependency of the network on local noise. This value was chosen to maximize the training set accuracy, as shown on Supplementary Fig. S1. A ROI comprising only brain pixels was manually drawn on one frame for each animal and used to mask out fUS information outside the brain, though this information can be relevant (Willis circle). The corresponding pixels were set to the minimum fUS value across the whole acquisition.
- ROI-based approach: the network takes as an input the mean fUS values in a number of predefined ROIs copied on all frames, representing known brain structures as described above.

For each acquisition (i.e. continuous imaging of one animal at a given probe position), a dataset comprising the same number of frames for each output category (i.e. for each state) was built for both ROI- and pixel-based classifications.

Additional analysis was carried out to determine the importance of information leakage from training to testing frames. For this purpose, the accuracy of the prediction was tested for each fold and each animal on a dataset comprising all frames not used in training or validation. Histograms of the average accuracy across folds/animals relative to the distance of the considered frames to the closest training frame were then obtained and are shown in the supplementary materials (Supplementary Fig. S4).

All fUS frames were normalized by the maximum and range of values in the image, to span the interval [− 1,1].

#### FCNN training

The networks were then trained on a random selection of 70% of the balanced dataset generated as described above, with the remaining frames used to build separate validation and testing datasets of the same size. This led to training datasets of 454–1822 frames and 785–870 frames for Sleep/Wake and locomotion state identification respectively, and testing datasets of 151–607 frames and 262–290 frames respectively. These values are reported in Supplementary Table S4.

All networks were trained using minibatch stochastic gradient descent, for a maximum of 10,000 epochs. The cost function used was

$$J=\frac{1}{2m}\left[\sum_{i}^{m}{\left({h}_{\theta }\left({x}^{i}\right)-{y}^{i}\right)}^{2}+\lambda \sum_{j}^{n}{\theta }_{j}^{2}\right]$$

where m is the number of samples, h_ϑ_(x^i^) is the network output for the input sample x^i^, y^i^ is the true label corresponding to sample x^i^, λ is the regularization parameter, n the number of layers and ϑj the matrix of network weights corresponding to layer j.

The training was stopped earlier if the network reached an accuracy higher than 98% on the validation dataset for at least 50 epochs. The learning rate α was gradually decreased using the formula: $\alpha =\frac{\alpha }{1+ep}$, with ep the number of the current epoch. No regularization was used (lambda = 0).

The initial value of the learning rate as well as the minibatch size were fine-tuned using cross-validation and the validation datasets, resulting in different optimal values according to the classification problems, as detailed below.

Accuracies were evaluated using k-fold cross-validation with a k value of 5, and stratified folds generated using the *cvpartition* function in Matlab from the statistics and machine learning toolbox. The absence of overfitting was ensured by visually checking that the network cost decreased throughout the training for both training and validation datasets. The significance of each prediction was evaluated using the permutation test, in the case of intra-animal decoding as well as inter-animal decoding. For each acquisition/model pair the accuracy of the prediction was compared to distribution of accuracies obtained for 1000 label permutations of the test data. The corresponding p-value was returned, with a significance level set at 0.05.

#### FCNN evaluation

The overall accuracy of each FCNN was calculated as the percentage of the testing set cases for which the network predicted the accurate label. The FCNN performance was further evaluated by calculating the precision P and recall R values for each sleep/wake state following the formula:

$$P=\frac{TP}{PredPos}= \frac{\% \, accurate}{\% \, classified},$$

$$R=\frac{TP}{Pos}= \frac{\% \, accurate}{\% \, ground \, truth}$$

where TP is the number of frames of that sleep/wake state accurately classified as such, PredPos is the number of frames classified by the algorithm as belonging to that sleep/wake state, and Pos is the number of frames actually belonging to that sleep/wake state.

The uncertainty in the classification (cf. Fig. 3) is calculated as 1 − (the difference between the probability output by the network for the class predicted, and the second highest probability output). It is used to illustrate the difficulty which the network has to classify a given frame. Note that this probability is given by the network output, i.e. before selecting as prediction the category with the highest probability.

When evaluating the decoding accuracy on a previously unseen animal, one region-based network was trained for each animal available. The same regions were used as inputs for all animals, making it possible to apply the weights learnt by one network to a different animal. The accuracy (as defined above) of the network generated for each animal was evaluated when applying it to each of the other animals. The accuracy reported is the average of accuracies obtained for each network applied to a different animal, i.e. 30 instances for 6 different animals available. This average accuracy is compared to the average accuracy obtained intra-animal, i.e. when evaluating the network trained on one animal on previously unseen frames of the same acquisition. This amounts to 6 instances for 6 animals available. The difference is named “accuracy lost” on Fig. 3.

#### FCNN importance maps

In order to understand the rules learnt by the networks, we further applied a visualization technique based on the Holdback Input Randomization method^23^ which provides a quantification of the importance of each pixel to the classification. This is done by fixing the network’s learned weights and running the network on a test given dataset with the input corresponding to a given pixel or ROI replaced by random values between -1 and 1. The method then evaluates the accuracy lost by the network when information about this particular input was suppressed, i.e. the importance of this input to the classification. This could be applied to both pixel-based (downsampled with a 2 × 2 kernel) and ROI-classifications, and the importance to classification was evaluated specifically for each category as the drop in the probability of each state output by the network in this specific category when this input was suppressed.

Importance maps for pixel-based classification were generated by reshaping the importance of the different inputs to match the original image dimensions. For region-based classification, this was done by assigning each importance value to a mask of the corresponding region in 2D.

## Supplementary Information

Supplementary Information 1.

Supplementary Information 2.

Supplementary Video 1.
