# Supplementary material for: Decoding behavior from global cerebrovascular activity using neural networks
Source: Sci Rep. 2023 Mar 2;13:3541. doi: 10.1038/s41598-023-30661-5 (PMC9981746; doi:10.1038/s41598-023-30661-5)
Supplement: Supplementary file 1 — Supplementary Information 1. [file 41598_2023_30661_MOESM1_ESM.pdf]

Decoding behavior from global cerebrovascular activity using neural networks

Béatrice Berthon[*,**,1], Antoine Bergel[**,1], Marta Matei[1], Mickaël Tanter[1]

1. Physics for Medicine Institute, INSERM U1273, CNRS UMR 8063, ESPCI Paris, PSL Research University

***Joint co-authors*

Supplementary material

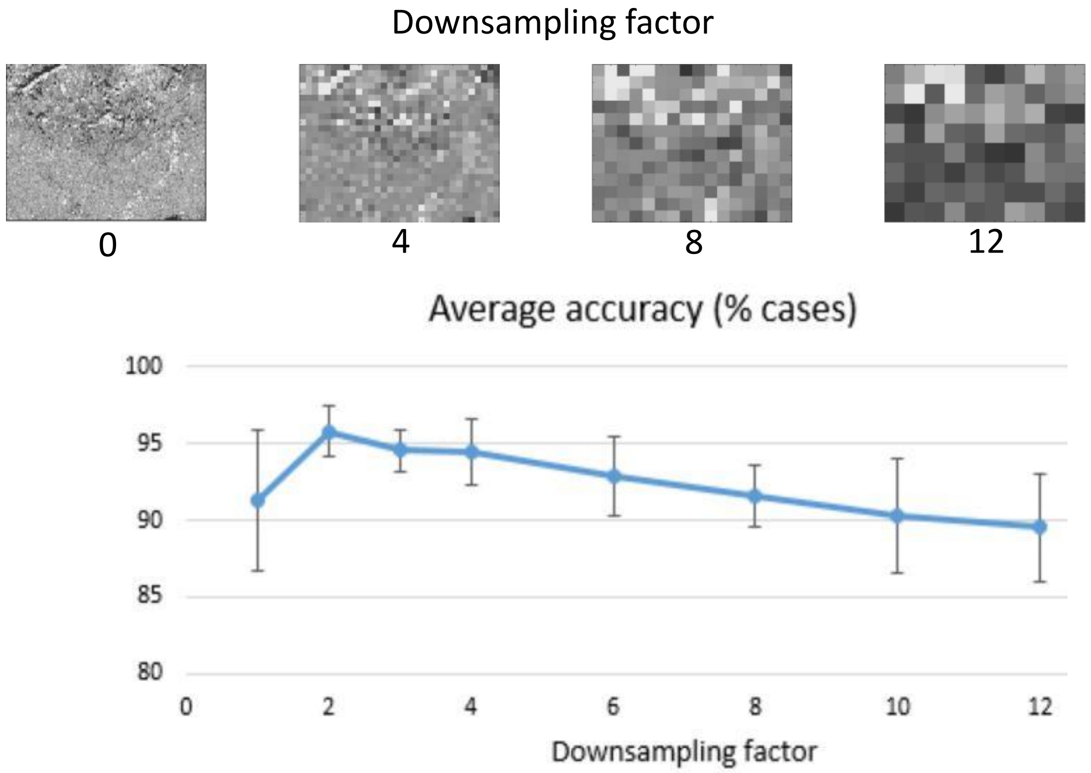

Figure S1. Evaluation of the average accuracy obtained for different downsampling factors of the original fUS images for pixel-wise classification. A downsampling value of 2 was selected as the best trade-off between image size and classification accuracy. The top row provides an example of CBV map displayed for the different downsampling factors indicated above the images.

			Movement		
			Accuracy (%)	P (%)	R (%)
Pixels	nΔCBV	average across animals	**98,0**	**98,7**	**97,1**
		min	**97,8**	**98,3**	**94,6**
		max	**98,5**	**99,0**	**98,4**
		average SD across instances	1,2	0,7	1,1
		min	0,4	0,4	0,6
		max	1,9	1,2	1,6
		Permutation test p-values range (number of non-significant results)	0,001 – 0,001 (0)		
	CBV	average across animals	**84,8**	**83,1**	**91,4**
		min	74,1	62,0	**91,0**
		max	**92,3**	**94,9**	**91,9**
		average SD across instances	1,3	2,7	2,6
		min	0,7	0,1	0,6
		max	2,4	7,6	6,6
		Permutation test p-values range (number of non-significant results)	0,001 – 0,001 (0)		
Regions	nΔCBV	average across animals	**92,7**	**92,1**	**88,9**
		min	**89,1**	**88,0**	**82,2**
		max	**95,3**	**95,6**	**94,4**
		average SD across instances	0,8	0,6	0,7
		min	0,6	0,4	0,6
		max	0,9	0,7	0,8
		Permutation test p-values range (number of non-significant results)	0,001– 0,008 (0)		
	CBV	average across animals	78,7	73,7	**82,9**
		min	76,4	63,4	71,6
		max	**81,5**	**80,2**	**92,6**
		average SD across instances	6,3	3,7	7,6
		min	1,6	0,7	0,4
		max	13,8	8,7	20,0
		Permutation test p-values range (number of non-significant results)	0,001 – 0,06 (1)		

Table S1. Intra-animal performance of the different networks trained to decode the locomotion state. Accuracy, Recall and Precision are provided as a % of cases, and are averaged across animals (6 different acquisitions) and folds (5 folds). The range of average and standard deviation (SD) is also provided. Values above 80% are highlighted in bold. The table also reports the range of p-values across cases (folds/animals) obtained by the permutation test checking for the significance of the prediction returned (significant p-value p<0.05), and the number of non-significant cases. The number of non-significant predictions across acquisitions/folds is indicated in brackets.

			Accuracy (%)	REMS P (%)	REMS R (%)	AW P (%)	AW R (%)	NREMS P (%)	NREMS R (%)	QW P (%)	QW R (%)
Pixels	nΔCBV	average across animals	**87,4**	**93,3**	**92,4**	**87,2**	**83,8**	**89,8**	**93,0**	**83,2**	**84,1**
		min	79,5	**87,1**	78,5	57,9	68,0	**83,7**	**89,7**	61,4	52,5
		max	**94,9**	**99,1**	**99,0**	**95,8**	**95,6**	**97,7**	**98,8**	**96,1**	**96,6**
		average SD across instances	4,0	3,4	2,6	5,4	7,9	4,0	2,2	3,7	4,5
		min	0,9	0,6	0,5	1,1	1,2	0,9	0,3	1,5	0,9
		max	8,8	12,9	7,4	16,3	20,5	10,6	4,4	6,6	9,1
		Permutation test p-values range (number of non-significant results)				0,001 – 0,001 (0)					
	CBV	average across animals	**83,0**	**94,6**	**94,9**	**84,5**	**83,1**	**84,5**	78,9	**82,7**	**87,5**
		min	72,6	**86,3**	75,4	73,3	70,8	70,2	60,0	72,8	65,9
		max	**94,3**	**100,0**	**100,0**	**96,4**	**98,2**	**94,5**	**93,7**	**90,7**	**100,0**
		average SD across instances	6,7	3,1	3,9	7,4	9,5	8,1	11,2	6,9	4,5
		min	0,9	0,0	0,0	0,5	1,0	0,8	1,5	2,5	0,0
		max	15,2	10,5	20,1	21,7	21,5	14,1	21,1	13,1	12,9
		Permutation test p-values range (number of non-significant results)				0,001 – 0,001 (0)					
Regions	nΔCBV	average across animals	75,2	**87,0**	**86,8**	72,9	74,5	76,9	77,4	71,0	68,7
		min	67,1	74,5	73,5	59,9	66,2	66,6	59,8	62,5	54,3
		max	**86,8**	**94,0**	**95,3**	**83,7**	**87,9**	**93,0**	**88,3**	**79,9**	**80,7**
		average SD across instances	1,2	1,1	1,1	2,0	1,5	1,4	1,1	1,8	3,2
		min	0,6	0,8	0,7	0,6	0,8	0,7	0,5	0,8	1,1
		max	2,2	2,4	2,0	4,0	1,8	3,7	1,5	2,8	6,6
		Permutation test p-values range (number of non-significant results)				0,001 – 0,001 (0)					
	CBV	average across animals	54,2	**84,8**	**91,7**	59,6	49,1	45,0	48,8	53,2	52,5
		min	47,4	74,3	76,8	45,6	34,0	29,9	15,0	40,4	19,3
		max	59,7	**98,6**	**100,0**	78,4	71,2	57,9	77,4	64,1	**82,7**
		average SD across instances	3,4	3,0	1,8	6,4	9,6	4,3	7,6	4,7	5,5
		min	0,4	0,3	0,0	0,8	1,0	0,7	0,6	0,2	0,9
		max	6,9	9,2	7,1	15,3	23,3	11,0	22,2	14,2	12,8
		Permutation test p-values range (number of non-significant results)				0,001 – 0,004 (0)					

Table S2. Intra-animal performance of the different networks trained to decode the sleep/wake state. Accuracy, Recall and Precision are provided as a % of cases, and are averaged across animals (6 different acquisitions) and folds (5 folds). The range of average and standard deviation (SD) is also provided. Values above 80% are highlighted in bold. The table also reports the range of p-values across cases (folds/animals) obtained by the permutation test checking for the significance of the prediction returned (significant p-value p<0.05). The number of non-significant predictions across acquisitions/folds is indicated in brackets.

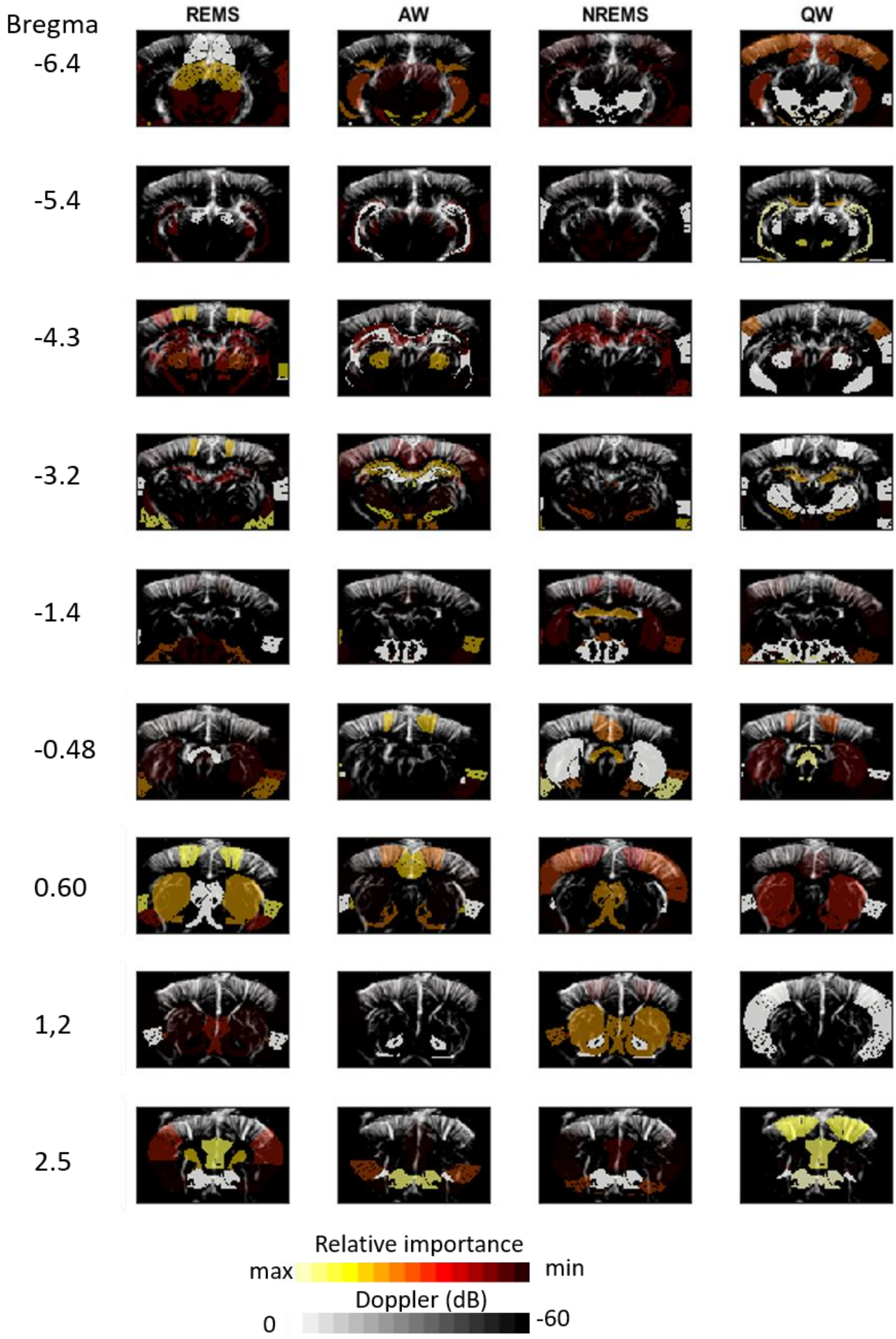

Figure S2. ROI importance maps for the sleep/wake state decoding for 9 coronal planes of the same animal. Each map is normalized by its maximum intensity value.

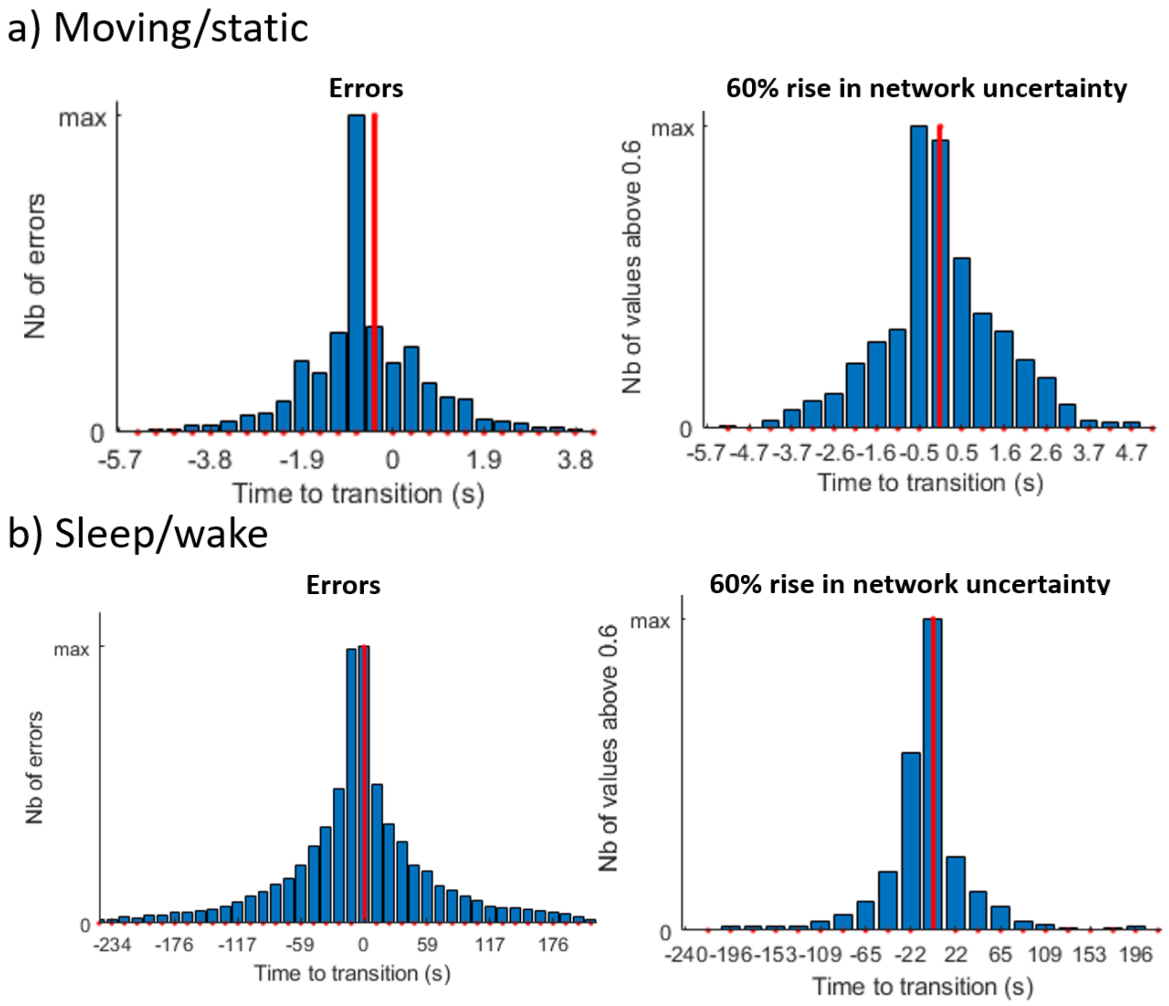

Figure S3. Number of decoding errors (left) and number of uncertainty values above 60% (right) versus the time to the nearest transition a) for movement and b) for sleep/wake state identification. Red vertical bars indicate the time of the state transition as defined by the labels.

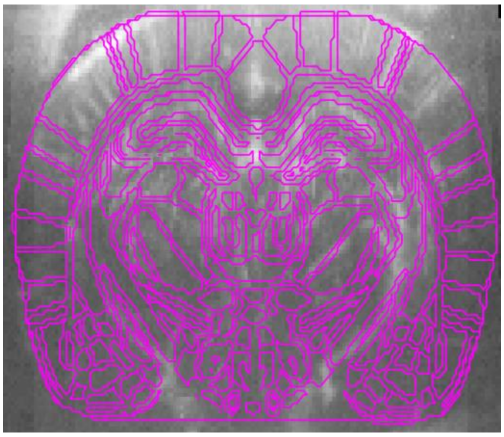

Description	Acronym	Corresponding Paxinos regions
Orbital Cortex	OCx	MO VO LO DLO
Limbic Cortex	LCx	PrL DP IL FR3
Cingulate Cortex	CCx	Cg Cg1 Cg2
Insular Cortex	ICx	DI GI AIP AID AIV
Motor Cortex	MCx	M1 M2
Somatosensory Cortex	SCx	S1 S1BF S1Tr S1HL S1FL S1J S1ULp S1Sh S1DZ S1DZO S2
Piriform Cortex	PiCx	DEn IEn VEn Pir1 Pir2 Pir3 APir3 APir RAPir RAPir3
Retrosplenial Cortex	RSCx	RSD RSGb RSGa RSGc
Parietal Cortex	PtCx	PtPR PtPC PtPD LPtA MPtA
Auditory Cortex	AuCx	Au1 AuV AuD TeA
Rhinal Cortex	RhCx	Ect PRh
Entorhinal Cortex	ECx	DLEnt DIEnt VIEnt MIEnt Ment MEntR CEnt
Visual Cortex	VCx	V1 V1B V1M V2L V2MM V2ML
Septum	Sp	TS MS LSI LSI1 LSI2 LSI3 LSI4 LSD LSV Ld PLd Shi Shy StHy vhc
Dentate Gyrus	DG	MoDG PoDG GrDG
CA1 region	CA1	CA1 OrCA1 PyCA1 RadCA1
CA2 region	CA2	CA2 OrCA2 PyCA2 RadCA3
CA3 region	CA3	CA3 OrCA3 PyCA3 RadCA3 SLuCA3
Subiculum	S	PrS PaS VS DS Post MoS STr
Fimbria	Fi	SFi fi
Residual Hippocampus	RHpc	Or Py Rad SLu LMol
Claustrum	Cl	DCl VCl Cl
Striatum	CPu	LSS CPu GP
Basal Forebrain	BF	HDB VDB VP cp EP B IPAC IPACL IPACM Nv mfb PIF IF IPL ICj SIB Acb AcbC AcbSh LAcbSh
Substrantia Nigra	SN	SNR SNL SNCD SNCV
Superior Colliculus	SC	SuG Op DpG InWh InG DpWh Zo bsc
Inferior Colliculus	IC	Com
Dorsal Périaqueductal gray	DPAG	PAG PlPAG LPAG DLPAG DMPAG
Ventral Périaqueductal gray	VPAG	VLPAG p1PAG Dk 3N InC Su3 Lth
Dorsal thalamus	DTh	LDVL LDDM AVVL AVDM AMV PT AD IAD LPLC LPLR LPMR LPMC OT DMDL CL CM PC PVA PV PVP AM IMA IMD MD MDC MDM PF RPF str AngT OPC
Ventral Thalamus	VTh	PoT Po VPL VPM VPPC VA VL VM VA/VL Re VRe Rh PaXi SubD SubV Sub Xi Eth REth sm eml PIL ml PP RRe Sc
Habenulla	Hb	LHb LHbM MHb LHbL Hb
Zona Incerta	ZI	ZI ZIC ZIR ZID ZIV SubI
Pretectal Nuclei	PN	PPT OPT MPT APTD APTV
Geniculate Nuclei	GN	DLG MGV MGD MZMG MGM bic SubG SubB VG VG1 VG2 VG3 IGL SG
Reticular Formation	Rt	Rt mRt p1Rt

Anterior Amygdala	AA	CxA1 CxA2 CxA3 AA ACo MeAD BMA I BLA CeM CeC EAC EAM EA MeAV CeL BLV LaDL IM STIA MePD IMG LaVL LaVM AHiAL MePV ASt
Posterior Amygdala	PA	PMCO APir AHiPM PLCo BLP BMP AHiPL LA
Dorsal Hypothalamus	DHy	RMC RPC STh scp PaR F A13 A1 A11 PaLM PaMM PaAP PaMP PaPo PaV SPF DA RI SPFPC fr CLi SpA Stg PHA
Lateral Hypothalamus	LHy	rs PH PLH PeFLH Pe PeF AVPe PHD JPLH MCLH TuLH
Ventral Hypothalamus	VHy	AH AHA AHC AHP LA ANS DMC DMD DMV VLH VMH VMHC VMHSh VMHDM VMHVL Arc ArcD ArcL ArcMP ArcM ArcLP RRF tth Te
Ventral Tegmental Area	VTA	VTAR PBP PBPI PBPm PN vtgx
Preoptic Area	PO	MCPO LPO MPOL MPOM MPA VOLT MnPO PDPO VLPO ESO SO MPO MPOC VMPO
Mammillary Nuclei	MMN	SuM SuMM SuML LM ML MM MnM DTM VTM PMV PMD mt smt pm mp
Olfactory Nuclei	ON	MTu Tu1 Tu2 Tu3 LOT1 LOT2 LOT3 AOVP AOP AOM AOD AOL AOV AOE GrO DTT1 DTT2 DTT3 lo
Cerebellum	Cb	Cb Fl
BrainStem	BS	RLi MnR PnR PnC Pn PnV 8n VCA s5
Pineal Gland	Pi	Pi
Striata Terminalis	ST	st STIA STMPM STMAL STMAM STLV STLI STMA STMPL STMPI STLD PS
Optic Chiasm	Och	opt SCh SChVM SChDL RCh RChL sox och InCSh LT mlf
Corpus Callosum	CC	alv fmj fmi dcw dhc ic f cc cg csc pc PrC Pcom ac acp aca MCPC cbw
Ventricles	V	D3V 3V LV chp IVF MRe Aq E
Vessels	ves	uppervessel lowervessel ach azac acer mcer basalvessel vessel sinus lhia anteriorvessel

Figure S4. Example of registration with the Paxinos atlas shown on a coronal plane. The table provides the correspondence between Paxinos Atlas regions and ROIs used in this paper.

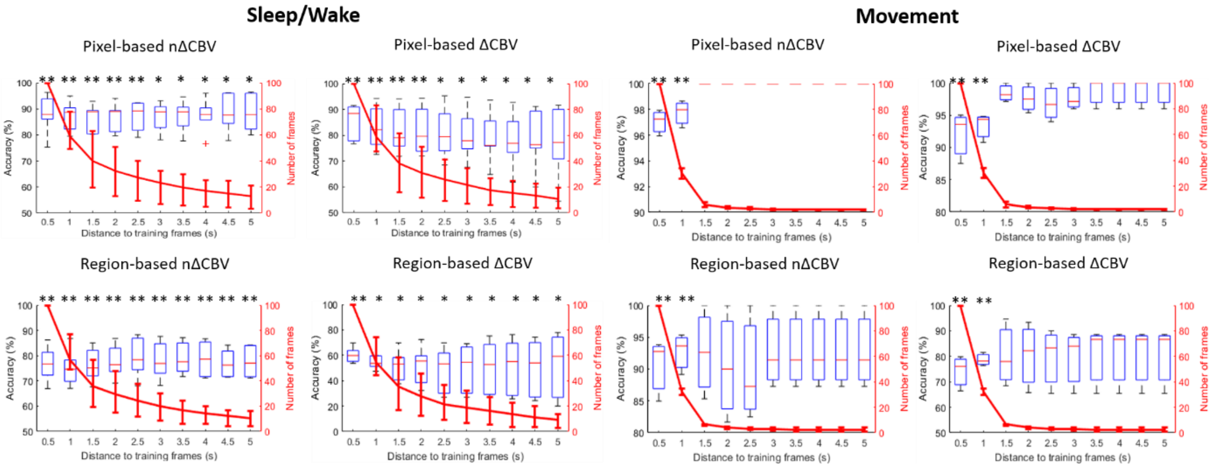

Figure S5. Analysis of the average accuracy across folds and acquisitions with respect to the time distance between the frames considered and the closest training frame. The box on the left of each plot represents the average accuracy calculated with all frames of the test sets. The next boxes on the right represent the average accuracy calculated for all frames of the test sets further than a certain temporal distance from the training frames. The error bars represent the highest and lowest accuracy values obtained across acquisitions/folds. The red line represents for each box the proportion of test frames remaining in the corresponding test set, averaged over the recordings and with error bars corresponding to the range of values obtained. Finally double asterisks (**) are used to mark bars for which all acquisitions/folds returned significant predictions according to the permutation test (p-value <0.05), and single asterisks (*) the bars for which more than half of the acquisitions/folds returned significant predictions. No permutation tests were run for Movement decoding when removing more than directly adjacent frames (i.e. box plots further than second from left), because of a lack of available frames.

Sleep/Wake					
**Recording N°**	**Full dataset**	**Balanced dataset**	**Training**	**Test**	**Test without directly adjacent frames**
**1**	3380	756	454	151	83
**2**	4752	1540	924	308	161
**3**	14308	3036	1822	607	339
**4**	8931	2028	1286	371	192
**5**	11935	1851	1111	370	230
**6**	7167	2529	1517	506	249

Locomotion					
**Recording N°**	**Full dataset**	**Balanced dataset**	**Training**	**Test**	**Test without directly adjacent frames**
**1**	1953	1308	785	262	89
**2**	1961	1450	870	290	76
**3**	1967	1432	859	286	89

Table S3. Summary of the number of frames obtained for each recording in the different datasets used.
