# Supplementary material for: Decoding behavior from global cerebrovascular activity using neural networks
Source: Sci Rep. 2023 Mar 2;13:3541. doi: 10.1038/s41598-023-30661-5 (PMC9981746; doi:10.1038/s41598-023-30661-5)
Supplement: Supplementary file 2 — Supplementary Information 2. [file 41598_2023_30661_MOESM2_ESM.docx]

Visualization of the network’s classification and errors in time and space during one sleep/wake acquisition. The top panel shows a hypnograph of the animal’s sleep/wake state at each time point of the acquisition. The bottom panel shows the network hidden neuron’s activation in the latent space in 3D, at each time point of the acquisition as a grey line, here in the case of sleep/wake state identification based on pixel values. Errors in the classification are displayed as a red circle. This visualization illustrates the similarities between fUS frames identified by the network by forming different clusters in this space, and shows the network clustering and errors throughout the whole acquisition as a temporal sequence.
